# Pre-natal Attachment and Parent-To-Infant Attachment: A Systematic Review

**DOI:** 10.3389/fpsyg.2021.620942

**Published:** 2021-03-17

**Authors:** Tommaso Trombetta, Maura Giordano, Fabrizio Santoniccolo, Laura Vismara, Anna Maria Della Vedova, Luca Rollè

**Affiliations:** ^1^Department of Psychology, University of Torino, Torino, Italy; ^2^Department of Pedagogy, Psychology, Philosophy, University of Cagliari, Cagliari, Italy; ^3^Department of Clinical and Experimental Sciences, University of Brescia, Brescia, Italy

**Keywords:** pre-natal attachment, parent-to-infant attachment, systematic review, pregnancy, post-partum

## Abstract

During the perinatal period, the establishment of the attachment relationship with the fetus and subsequently with the real child is crucial for the parents' and the child's well-being. Coherently with the assumption that the attachment relationship starts to develop during pregnancy, this systematic review aims to analyze and systematize studies focused on the association between pre-natal attachment and parent-to-infant attachment, in order to clarify the emerging results and provide useful information for clinical purposes. Nineteen studies were included. Sixteen researches identified a positive relationship between pre-natal attachment and parent-to-infant attachment, and three articles highlighted a negative association between antenatal attachment and post-partum bonding disorders. These results were found both in women and men, in normative and at-risk pregnancies, adopting different assessment approaches (i.e., self-report measures, observations, and projective measures). However, only small or moderate associations were found. Future studies are needed to further confirm these findings across different populations (e.g., male samples, non-normative samples or samples in disadvantaged conditions) and with different methodological approaches (e.g., observational measures). Moreover, studies would be needed in order to clarify mechanisms through which pre-natal attachment influences parent-to-infant attachment, as well as protective and risk factors which intervene between these two variables.

## Introduction

The transition to parenthood is a critical period of adjustment for both men and women, where the attainment of the parental role plays a major part in the well-being of the family as well as in the development of the child. During this period, the establishment of an affective tie with the fetus and subsequently the newborn is a major goal that can impact on the child's health and psychological development (Wilson et al., 2000; Laxton-Kane and Slade, 2002; Della Vedova, 2005; Della Vedova et al., 2008; Branjerdporn et al., 2017).

In literature, the term “attachment,” proposed by Bowlby (1969), refers to the relationship that the child develops with a caregiver aiming to obtain a secure base from which to explore the world and, if it is necessary, proximity, protection, and a retreat for safety and comfort in conditions of stress and danger (Ainsworth, 1969; Bowlby, 1969; Ainsworth et al., 1978; Waters and Cummings, 2000). During the first relational experiences, the child develops *internal working models* (i.e., mental images of the self, others and the relationships) which are the basis of later attachments in life (Bowlby, 1982; Benoit, 2004) and influence affect regulation strategies and cognitions (Brennan and Shaver, 1995; Shaver and Mikulincer, 2002; Mikulincer et al., 2003; Schore and Schore, 2008). According to the “attachment theory” (Bowlby, 1969), at the end of the 1st year of life the child has formed a specific style of attachment to his caregivers. The child's attachment style will be structured as “secure” or “insecure” depending on the quality of early relational experiences. If the baby enjoys a nurturing and safe relationship with those who care for him/her, the baby will gain a secure personal basis and therefore a sense of trust in others. Thus, the complementary parental side of attachment, which Bowlby defined the “caregiving system,” is an indispensable condition for the healthy development of the child (Buist et al., 2003; Righetti and Sette, 2008; Habib and Lancaster, 2010; Della Vedova et al., 2011; Premberg et al., 2011; Velotti et al., 2011; Prino et al., 2016; Vismara et al., 2016; Rollè et al., 2017; Ionio et al., 2019; Riva Crugnola et al., 2020).

Ainsworth et al. (1978) identified four dimensions of maternal behaviors which are connected with the security of the infant attachment: sensitivity, acceptance, cooperation and accessibility.

Other researchers (van IJzendoorn, 1995; Miljkovitch et al., 2004; Arnott and Meins, 2007; Korja et al., 2010; Waters et al., 2018) underlined that parental caregiving strategies are influenced by a range of conscious and unconscious factors (i.e., internal working model or attachment's representation) based on first attachment's experiences to their own caregivers. Within this approach, parental attachment's representations of first attachment's experiences influence adult attachment style and affect the sensitivity with which parents respond to their infant's needs (Main et al., 1985; van IJzendoorn, 1992). Therefore, the parental side of attachment is a fundamental condition for the child's development, and, as many authors from a psychoanalytical domain suggested since the '40 (Deutsch, 1945; Winnicott, 1956; Bibring, 1959), it is supposed to start in the mother (and father's) mind during pregnancy. Along with this framework, the interest of clinicians and researchers turned to the pre-natal phases of the relationship, considering the parental affective investment toward the fetus as a precursor of the post-natal relationship. Authors who pioneered this area of research have used the term “attachment,” from Bowlby's theory, to describe the parental emotional bonding with the fetus which gradually develops over the course of pregnancy (*pre-natal attachment*), extending this concept also to the post-natal period (*parent-to-infant attachment*). Even though the use of the word attachment to indicate the parental emotional involvement toward the fetus/baby is questionable (Walsh, 2010), pre-natal/post-natal attachment has become commonly used to indicate the affectionate tie that parents develop toward their unborn baby.

Around the '80 a theoretical construct has been created with the aim to systematically investigate the affective investment of the parents toward their fetus. Cranley (1981), on the basis of her experience in the midwifery field, proposed the construct of “pre-natal attachment” according to the psychoanalytical literature of maternal adjustment to pregnancy and role attainment (Deutsch, 1945; Benedek, 1959; Bibring, 1959) and created a specific questionnaire to evaluate its characteristics. The Maternal-Fetal Attachment Scale (MFAS; Cranley, 1981), consists of five subscales: differentiation of self from the fetus; interaction with the fetus; attributing characteristics and intentions to the fetus; giving of self; role taking. The measure also includes a parallel form assessing the paternal fetal attachment (PFAS; Weaver and Cranley, 1983). Müller (1993, 1996), in order to measure the special affectionate relationship that develops between a woman and her fetus, developed the 21-item Pre-natal Attachment Inventory (PAI, 1993) achieved identifying four themes: Preparedness, Fantasizing, Affection, and Interaction. Within this perspective, Condon (1993), starting from the idea that maternal emotional investment toward the fetus must be distinguished from features of maternal pregnancy adaptation and maternal role attainment, reformulated the pre-natal attachment construct. He proposed a model that gives attention to “the emotional bond or tie of affection experienced by the parent toward the infant” (Condon and Corkindale, 1998; p. 57), which from the authors' perspective, promotes dispositions to caregiving that can be subsequently translated into overt behaviors oriented toward the child (i.e., information seeking, proximity, protection, pleasing, and gratifying). The authors defined this tie of affection as parent-to-fetus attachment in pregnancy and parent-to-infant attachment in the post-natal period, and identified four indicators of the construct: pleasure in proximity, tolerance, need gratification and protection, knowledge acquisition (Condon and Corkindale, 1998; Scopesi et al., 2004; Condon et al., 2008). To measure the construct, Condon created two questionnaires for mothers and fathers: the Maternal Antenatal Attachment Scale and the Paternal Antenatal Attachment Scale (MAAS, PAAS; Condon, 1993) for the pre-natal period, and the Maternal Post-natal Attachment Scale and Paternal Post-natal Attachment Scale (MPAS, PPAS; Condon and Corkindale, 1998) for the post-natal period.

In the last decades, the terms “parent-infant bonding” or “post-partum bond” were often used as synonyms of “parent-to-infant attachment” (Brockington et al., 2006; Altaweli and Roberts, 2010; Bicking Kinsey and Hupcey, 2013). The concept of “bonding” or “bond” has been initially defined by Klaus and Kennell (1976) as a parent-child tie based on skin-to-skin contact during the early sensitive post-partum period. The authors Klaus and Kennell (1982); (Kennell and Klaus, 1998) later criticized their definition as it did not consider the affective domain. According to this, recent literature describes the term “bonding” or “bond” as an affective tie that the parent develops toward the child (Kennell and McGrath, 2005; Taylor et al., 2005; Bicking Kinsey and Hupcey, 2013).

Although, as previously noted, “parent-to-infant attachment” and “parent-infant bonding” has been frequently used as synonyms, nonetheless, regardless of the definition adopted, the affective tie experienced by parents toward the child is the main focus (Bicking Kinsey and Hupcey, 2013).

Therefore, for convenience, we will refer to this specific affective tie in terms of parent-to-infant attachment, independently of the term used in the included studies.

Differences emerged on the assessment approach used to evaluate parent-to-infant attachment and thus on its operationalization (see Table 1), which may influence the obtained results and make it difficult to compare the conclusions highlighted across the studies [for an extensive review see Wittkowski et al. (2020)]. While Condon and Corkindale's MPAS and PPAS (1998), as other self-report instruments - e.g., the MIBS (Taylor et al., 2005) and the PPBS (Cuijlits et al., 2016) - emphasized the affective and cognitive dimension of this construct, focusing on the subjective parental experience of parent-to-infant attachment, other authors assessed it resorting to observational approaches based on behavioral indicators (e.g., Avant, 1982), in line with a large body of literature on infant-to-parent attachment, or projective methods (e.g., van Bakel et al., 2013). As stated by Condon and Corkindale (1998), attachment behaviors have to be considered as possible but not necessary consequences of the attachment experience. In line with this consideration and highlighting the methodological biases related to observational approaches, these authors suggest assessing parent-to-infant attachment administering self-report measures, more capable to capture the subjective experience and the core of the attachment bond (i.e., love), which can in turn promote parental dispositions to caregiving. Nonetheless, a combination of observational and self-report measures would be ideally recommended (Condon and Corkindale, 1998; Condon, 2012).

**Table 1:** Measurement of Parent-to-Infant Attachment.

**Measurement**	**References**	Construct assessed^*^	**Procedure**	**Subscales**	**Dimensions explored/Sample items**
How I Feel About My Baby Now Questionnaire (FAB)	Leifer (1977)	Parent-to-infant attachment	Self-report 10 items	No subscales	Maternal and paternal feelings that represent affection toward the child are evaluated Sample items: positive statements (“I feel tenderly toward my baby”), negative statements (“I feel disinterested in my baby”) Response options: 1–4
Avant's questionnaire of mother-infant attachment behaviors	Avant (1982)	Mother-to-infant attachment	Observational approach In the first 30 s of each minute behaviors are observed, then they are recorded. Each behavior is recorded once per minute	Not applicable	Three groups of maternal behaviors (emotional, proximity, and caring behaviors) acts during interactions with the child are evaluated
Maternal Attachment Inventory (MAI)	Müller (1994)	Mother-to-infant attachment	Self-report 26 items	No subscales	Maternal activities and feelings that indicate affection are evaluated Sample items: “I feel love for my baby”; “I look forward to being with my baby” Response options: 1–4
Maternal Post-natal Attachment Scale (MPAS)	Condon and Corkindale (1998)	Mother-to-infant attachment	Self-report 19 items	Three subscales: 1. Quality of attachment 2. Absence of hostility 3. Pleasure in interaction	Maternal thoughts, feelings, and behaviors toward the child are evaluated Sample items: 1. Quality subscale (9 items, e.g., “Over the last 2 weeks I would describe my feelings for the baby as: dislike (1)–intense affection (5)”) 2. Absence of hostility toward the infant (5 items, e.g., “When I am caring for the baby, I get feelings of annoyance or irritation: very frequently (1)–never (5)”) 3. Pleasure in the interaction with the infant [5 items, e.g., “When I have to leave the baby: I usually feel rather sad (1)–I usually feel rather relieved (5)”] Response options: 1–5 (each item has a 2, 4, or 5 point response option)
Post-partum Bonding Questionnaire (PBQ)	Brockington et al. (2001)	Mother-to-infant attachment	Self-report 25 items	Four subscales: 1. Impaired bonding 2. Rejection and anger 3. Anxiety about care 4. Risk of abuse	Maternal feelings, cognition and behaviors experienced during interactions with the child are evaluated Sample items: 1. Impaired bonding (12 items, e.g., “The baby does not seem to be mine”) 2. Rejection and anger (7 items, e.g., “I feel distant from my baby”) 3. Anxiety about care (4 items, e.g., “My baby makes me feel anxious”) 4. Risk of abuse (2 items, e.g., “I have done harmful things to my baby”) Response options: 0–5
Mother-to-Infant Bonding Scale (MIBS)	Taylor et al. (2005)	Mother-to-infant attachment	Self-report 8 items	No subscales	8 adjectives that describe mother's feeling toward the infant are presented: loving, resentful, neutral or felt nothing, joyful, dislike, protective, disappointed, and aggressive
Post-partum Bonding Questionnaire−16 items (PBQ-16)	Reck et al. (2006)	Mother-to-infant attachment	Self-report 16 items	No subscales	Maternal feelings, cognition and behaviors experienced during interactions with the child are evaluated Sample items: “I feel distant from my baby”; “My baby irritates me”; “My baby is easily comforted” Response options: 0–5
Father-Infant Attachment Inventory (FIAI)	Hjelmstedt and Collins (2008)	Father-to-infant attachment	Self-report 26 items	No subscales	Paternal feelings that indicate affection are evaluated Sample items: “I feel love for my baby” and “I look forward to being with my baby” Response options: 1-4
Paternal Post-natal Attachment Scale (PPAS)	Condon et al. (2008)	Father-to-infant attachment	Self-report 19 items	Three subscales: 1. Patience and tolerance 2. Pleasure in interaction 3. Affection and pride	Paternal thoughts, feelings, and behaviors toward the child are evaluated Sample items: 1. Patience and tolerance (absence of annoyance/irritability, lack of resentment; 8 items, e.g., “When I'm looking after my baby, I feel sad, frustrated or irritated”) 2. Pleasure in interaction (satisfaction, competence, involvement, anticipation of the interaction; 7 items, e.g., “When I am with my baby, I feel impatient”) 3. Affection and pride (more stable feelings and cognitions as the sense of ownership, pride, and feelings of affection; 4 items, e.g., “In the last 3 months, I felt I have had no time for myself or to do things that I'm interested in”) Response options: 1–5 (each item has a 2, 4, or 5 point response option)
Pictorial Representation of Attachment Measure (PRAM)	van Bakel et al. (2013)	Parent-to-infant attachment	Projective method The question presented to the parents is: “Where would you place your newborn baby in your life right now?” Then, with a symbolic sticker, they place the Self of the baby in the sheet's space. The score is represented by the distance between the “Self” circle and the “Self-Baby” circle.	Not Applicable	Parental non-verbal representations of feelings of attachment and connectedness to the child
Pre-natal and Post-natal Bonding Scale (PPBS)	Cuijlits et al. (2016)	Mother-to-infant attachment	Self-report 5 items	No subscales	Maternal feelings toward the child are evaluated Sample items: “During the last four weeks, I could describe my feeling toward my baby the best as to be loving (item 1), happy and joyful (item 2)” Response options: 0–3

* *The construct assessed by each instrument has been defined as “parent/father/mother-to-infant attachment” irrespective of the definition provided by authors*.

Parent-to-infant attachment emerges as an important factor contributing to the quality of the subsequent reciprocal relationship, as well as to the well-being and the development of the child (Young, 2013; Parfitt et al., 2014). A stronger parent-to-infant attachment promotes the child's social, cognitive, and behavioral development (Schenk et al., 2005; Mason et al., 2011; Ip et al., 2018) as well as better mother-child interactions which in turn improve child's emotional regulation (Klaus and Kennell, 1982; Cigoli et al., 2006; Mason et al., 2011; Behrendt et al., 2018, 2019; Brake et al., 2020; Ponti et al., 2020). In addition, parents with higher levels of parent-to-infant attachment have higher responsiveness and sensitivity, which promote the development of a child's secure attachment and the infant's exploration of the environment maintaining an appropriate level of stimulation (Ainsworth et al., 1971; Solomon and George, 1996; Siddiqui and Hägglöf, 2000; Sandbrook and Adamson-Macedo, 2004; Blair et al., 2006; Rossen et al., 2019).

As stated by several authors (Deutsch, 1945; Winnicott, 1956; Benedek, 1959; Bibring, 1959; Rubin, 1967), the attachment bond starts to develop during pregnancy through the perception of the fetus as a human being with a separate self and specific needs. This relationship between the parent and the fetus begins in the parents' mind on imaginary level through the development of mental representations of the future child (Righetti, 2003; Ammaniti et al., 2006, 2012; Raphael-Leff, 2010; Vreeswijk et al., 2014). As pregnancy progresses, this attachment bond includes the imagined child, who is gradually experienced as a separate and differentiate human being through the contribution of fetal movements and fetal ultrasound (Ammaniti et al., 1992; Raphael-Leff, 2001; Della Vedova et al., 2008; Righetti and Sette, 2008; Vreeswijk et al., 2014). Indeed, during pregnancy, the fetus goes through an extraordinary motor and sensory development that allows him/her to perceive and be perceived, and to establish an early psychosomatic communication and first somatic memories (Borsani et al., 2019). Pre-natal attachment has been described as a bond between a parent and his/her unborn child consisting of mental representations, fantasies, emotions and mental capacities necessary to identify another human being (Cranley, 1981; Condon, 1993; Müller, 1993; Laxton-Kane and Slade, 2002; Doan and Zimerman, 2003). In line with the definition of parent-to-infant attachment (Condon and Corkindale, 1998), Condon (1993) described this bond as an experience of love expressed through the parental disposition to form an image of the baby, to interact with and protect the fetus while avoiding separation or loss and gratifying his/her needs.

The literature investigating pre-natal attachment was more focused on its predictors rather than its consequences (Damato, 2004; Cannella, 2005; Hjelmstedt et al., 2007; Bielawska-Batorowicz and Siddiqui, 2008; Yarcheski et al., 2009; Ossa et al., 2012; Della Vedova and Cristini, 2019; Tichelman et al., 2019). Nonetheless, several studies highlighted the predictive role of pre-natal attachment on the child's socioemotional, behavioral and cognitive development in early childhood and the parent's mental health during the perinatal period (Misri and Kendrick, 2008; Yarcheski et al., 2009; Alhusen et al., 2013; Della Vedova, 2014; Walsh et al., 2014; Branjerdporn et al., 2017; Cildir et al., 2019; Rollè et al., 2020).

In light of the above-mentioned literature, pre-natal attachment and parent-to-infant attachment appear as noteworthy factors for the quality of the subsequent parent-child reciprocal relationship and for their well-being. Coherently with the assumption that the development of the attachment bond starts during pregnancy, many studies explored the relation between these two variables (see Table 2). In order to clarify the emerging results, the current paper aims to review and systematize the papers focused on the association between pre-natal attachment and parent-to-infant attachment, providing information that can promote early interventions during the perinatal period, which can have long term impacts on relationship quality and familial well-being.

**Table 2:** Characteristics of the included researches.

**References**	**Title**	**Participants**	**Pre-birth evaluation tools**	**Pre-birth evaluation time**	**Post-partum evaluation tools**	**Post-partum evaluation time**	**Results and Conclusions**
Mercer and Ferketich (1994)	Maternal-Infant Attachment of Experienced and Inexperienced Mothers during Infancy	136 experienced mothers and 166 inexperienced mothers	MFAS	between 24th and 34th week of pregnancy	FAB	T1: post-partum hospitalization T2: 1st month post-partum T3: 4th month post-partum T4: 8th month post-partum	The results showed a positive association between maternal pre-natal attachment and mother-to-infant attachment only during post-partum hospitalization period (not at 1, 4, and 8 months post-partum) among inexperienced mothers, and during post-partum hospitalization, at 1 and 4 months post-partum (not at 8 months) in experienced mothers.
Ferketich and Mercer (1995)	Paternal-Infant Attachment of Experienced and Inexperienced Mothers during Infancy	72 experienced fathers and 93 inexperienced fathers	PFAS	between 24th and 34th week of pregnancy	FAB	T1: post-partum hospitalization T2: 1st month post-partum T3: 4th month post-partum T4: 8th month post-partum	A positive association was found between paternal pre-natal attachment and father-to-infant attachment during post-partum hospitalization, at 1 and 4 months post-partum (not at 8 months post-partum) in experienced fathers, and during post-partum hospitalization and at 1 month (not at 4 and 8 months post-partum) in inexperienced fathers.
Müller (1996)	Pre-natal and Post-natal Attachment: A Modest Correlation	196 women	PAI	2nd half of pregnancy	MAI FAB	between 1st and 2nd month after delivery	A positive association was found between the PAI and the MAI scores. Results showed also a positive but smaller correlation between the PAI and the FAB scores.
Damato (2004)	Pre-natal Attachment and other correlates of post-natal maternal attachment to twins	139 women	PAI	3rd trimester of the pregnancy	MAI	1st month after delivery	Antenatal attachment was positively associated with mother-to-infant attachment. The relation between the two variables was moderated by post-partum depression, method of delivery, and need for admission to the NICU.
Hjelmstedt and Collins (2008)	Psychological functioning and predictors of father–infant relationship in IVF fathers and controls	53 IVF men and 36 controls	PFAS	26th week of pregnancy	FIAI	2nd month post-partum	A positive association between fetal attachment and post-natal bonding emerged.
Condon et al. (2013)	A longitudinal study of father-to-infant attachment: antecedents and correlates	204 men	PAAS	3rd trimester of pregnancy	PPAS	6th month post-partum and 12th month post-partum	Paternal fetal attachment was positively associated with father–infant attachment at 6 and 12 months post-natally. Antenatal attachment predicted with 72% and 68% confidence which men will be in the upper and lower attachment quartiles at 6 months and at 12 months post-partum, respectively.
Dubber et al. (2015)	Post-partum bonding: the role of perinatal depression, anxiety and maternal–fetal bonding during pregnancy	80 women	MFAS	2nd trimester of pregnancy	PBQ-16	8th week after delivery	Pre-natal attachment was negatively associated with post-natal bonding impairments.
Taffazoli et al. (2015)	The Relationship between Maternal-Fetal Attachment and Mother-Infant Attachment Behaviors in Primiparous Women Referring to Mashhad Health Care Centers	100 women	MFAS	between 35th and 41st week of pregnancy	Avant's questionnaire of mother-infant attachment behaviors	T1: 4th week after delivery T2: 8th week after delivery	A positive association between maternal-fetal attachment, and emotional and proximity attachment behaviors was found at 4 and 8 weeks after delivery. No significant relation was found between maternal fetal attachment and mother-to-infant caring behaviors at 4 and 8 weeks after delivery.
Rossen et al. (2016)	Predictors of post-natal mother-infant bonding: the role of antenatal bonding, maternal substance use and mental health	372 women	MAAS	T1: 1st trimester of pregnancy T2: 2nd trimester of the pregnancy T3: 3rd trimester of pregnancy	MPAS	8th week after delivery	Pre-natal attachment, assessed during all three trimesters of pregnancy, was positively associated with mother-to-infant attachment at 8 weeks after delivery. The significant association between the two variables increased during pregnancy.
de Cock et al. (2016)	Continuous Feelings of Love? The Parental Bond from Pregnancy to Toddlerhood	322 women247 men	MAAS PAAS	26th week of pregnancy	MPAS PPAS	6th month and 24th month after delivery	A stability between pre-natal attachment and parent-to-infant attachment at 6 and 24 months post-partum emerged among both women and men.
Rossen et al. (2017)	Maternal Bonding through Pregnancy and Post-natal: Findings from an Australian Longitudinal Study	372 women	MAAS	T1: 1st trimester of pregnancy T2: 2nd trimester of pregnancy T3: 3rd trimester of pregnancy	MPAS	T4: 8th week after delivery	A positive correlation and a stability between the MAAS scores assessed at all trimester and the MPAS scores at 8 weeks post-partum emerged. A stability of the scores on the quality subscales of the MAAS and MPAS emerged through pregnancy and post-partum.
Luz et al. (2017)	Antenatal determinants of parental attachment and parenting alliance: how do mothers and fathers differ?	40 couples	MAAS PAAS	3rd trimester of pregnancy	MPAS PPAS	2nd month after delivery	Maternal and paternal antenatal attachment predicted father-to-infant attachment, while mother-to-infant attachment was only predicted by maternal pre-natal attachment.
Daglar and Nur (2018)	Level of mother-baby bonding and influencing factors during pregnancy and post-partum period	227 women	PAI	After the 35th week of pregnancy	MIBS	8th day after delivery	A positive correlation between pre-natal and post-natal bond was found.
Petri et al. (2018)	Maternal–fetal attachment independently predicts the quality of maternal–infant bonding and post-partum psychopathology	106 women	MAAS	6th month of pregnancy	MPAS	1st month after delivery	Higher levels of antenatal attachment independently predicted higher levels of mother-to-infant attachment.
Cuijlits et al. (2019)	Risk and protective factors for pre- and post-natal bonding	793 women	PPBS	T1: 32nd week of pregnancy	PPBS	T2: 8th month post-natally	There was a significant and positive association between pre-natal and post-natal bonding.
Fijałkowska and Bielawska-Batorowicz (2019)	A longitudinal study of parental attachment: pre- and post-natal study with couples	35 couples	MAAS PAAS PRAM	3rd trimester of pregnancy (between the 27th and the 40th week of pregnancy)	MPAS PPAS PRAM	Between the 2nd and the 8th week after childbirth	A positive correlation between pre-natal and post-natal attachment was found for both women and men, irrespective of the assessment tool administered.
Matthies et al. (2020)	Maternal–fetal attachment protects against post-partum anxiety: the mediating role of post-partum bonding and partnership satisfaction	166 women	MFAS	T1: 3rd trimester of pregnancy	PBQ	T2: 1st week post-partum T3: 4th month post-partum	Fetal attachment was negatively associated with post-natal bonding impairments at T2 and T3.
Smorti et al. (2020)	The mother-child attachment bond before and after birth: The role of maternal perception of traumatic childbirth	105 women	PAI	between the 31st and the 32nd week of pregnancy	MPAS	3rd month post-partum	Pre-natal attachment was positively associated with mother-to-infant attachment, both directly and indirectly, through the traumatic childbirth experience.
Zdolska-Wawrzkiewicz et al. (2020)	The Dynamics of Becoming a Mother during Pregnancy and After Childbirth	86 women	MFAS	Data retrieved during pregnancy	PBQ	Data retrieved after half a year after childbirth	Maternal pre-natal attachment was negatively associated with post-natal bonding impairments.

*MFAS, Maternal Fetal Attachment Scale; FAB, How I Feel About My Baby Now Questionnaire; PFAS, Paternal Fetal Attachment scale; PAI, Pre-natal Attachment Inventory; MAI, Maternal Attachment Inventory; FIAI, Father Infant Attachment Inventory; PAAS, Paternal Antenatal Attachment Scale; PPAS, Paternal Post-natal Attachment Scale; PBQ, Post-partum Bonding Questionnaire; MPAS, Maternal Post-natal Attachment Scale; MIBS, Mother-to-Infant Bonding Scale; PPBS, Pre- and Post-natal Bondings Scale; PRAM, Pictorial Representation of Attachment Measure*.

## Materials and Methods

### Data Source and Search Strategy

The current systematic review followed the Preferred Reporting Items for Systematic Review and Meta-Analysis (PRISMA) statement (Moher et al., 2009). Two independent reviewers searched through EBSCO databases (CINAHL Complete, Family Studies Abstracts, Mental Measurements Yearbook, PsycINFO, Social Sciences Abstracts—H.W. Wilson, Sociology Source Ultimate), PubMed, Scopus, and Web of Science (All Databases). They examined titles, abstracts, and full texts to detect eligible studies published until May 2020. The systematic search was performed using the following keywords: (“pre-natal attachment” OR “maternal fetal attachment” OR “parental fetal attachment” OR “paternal fetal attachment” OR “pre-partum attachment” OR “antenatal attachment” OR “pre-natal bond^*^” OR “maternal fetal bond^*^” OR “parental fetal bond^*^” OR “paternal fetal bond^*^” OR “pre-partum bond^*^” OR “antenatal bond^*^”) AND (“attachment” OR “bond^*^”).

### Inclusion and Exclusion Criteria

Papers were included if they were: (1) an original research paper, (2) published in English, and (3) focused on the association between pre-natal attachment and parent-to-infant attachment. Articles that did not match these inclusion criteria were excluded. Studies that assessed validity, reliability or psychometric properties of pre-natal or post-natal attachment instruments were excluded, considering that the analysis of the association between the two variables was not the main focus of these articles. Reviews and meta-analysis were excluded as well. No time restrictions for systematic searching were imposed: all the articles published until May 2020 were considered.

### Study Selection and Data Extraction

The search on EBSCO provided 1,016 results, 31 of which were selected for the full text review, after the screening of titles and abstracts. The search on PubMed yielded 461 results, 24 of which were selected. Web of Science produced 543 papers and 24 were selected. Scopus provided 2530 articles, 28 of which were selected. After the duplicates' removal, 35 articles were left and were reviewed in their full text.

**Figure 1:**
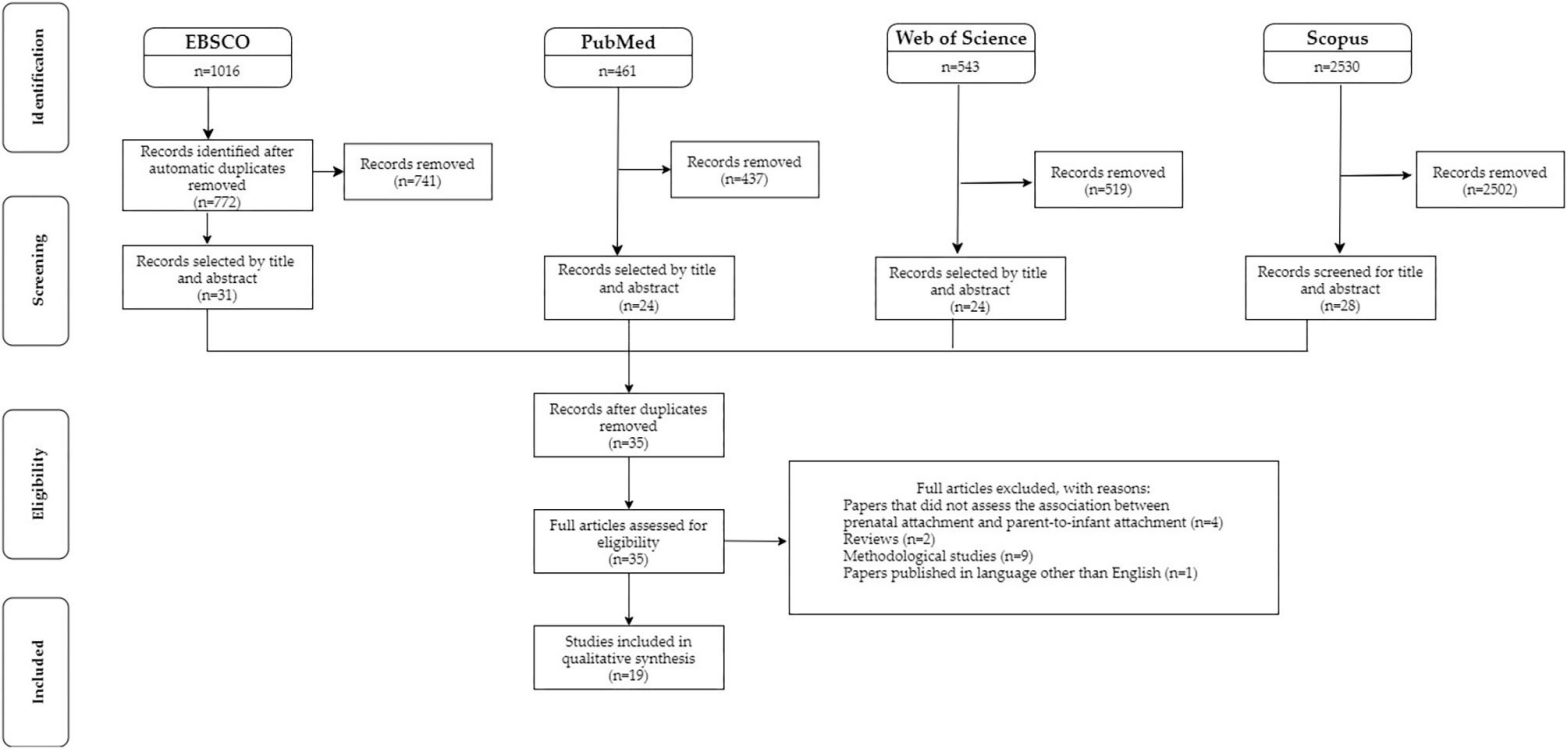
Flowchart of the selection procedure adapted from the Preferred Reporting Items for Systematic reviews and Meta-Analyses (PRISMA) (Moher et al., 2009).

Of these, 19 studies matched the inclusion criteria and were thus included in the current systematic review, while 16 studies were excluded for the following reasons: four articles did not assess the association between pre-natal attachment and parent-to-infant attachment; two were theoretical articles; nine were methodological studies; and one paper was published in a language other than English (Figure 1). Any disagreement between the two independent reviewers during the study selection and data extraction processes were discussed with a third reviewer, and a unanimous agreement was reached.

## Results

**Figure 2:**
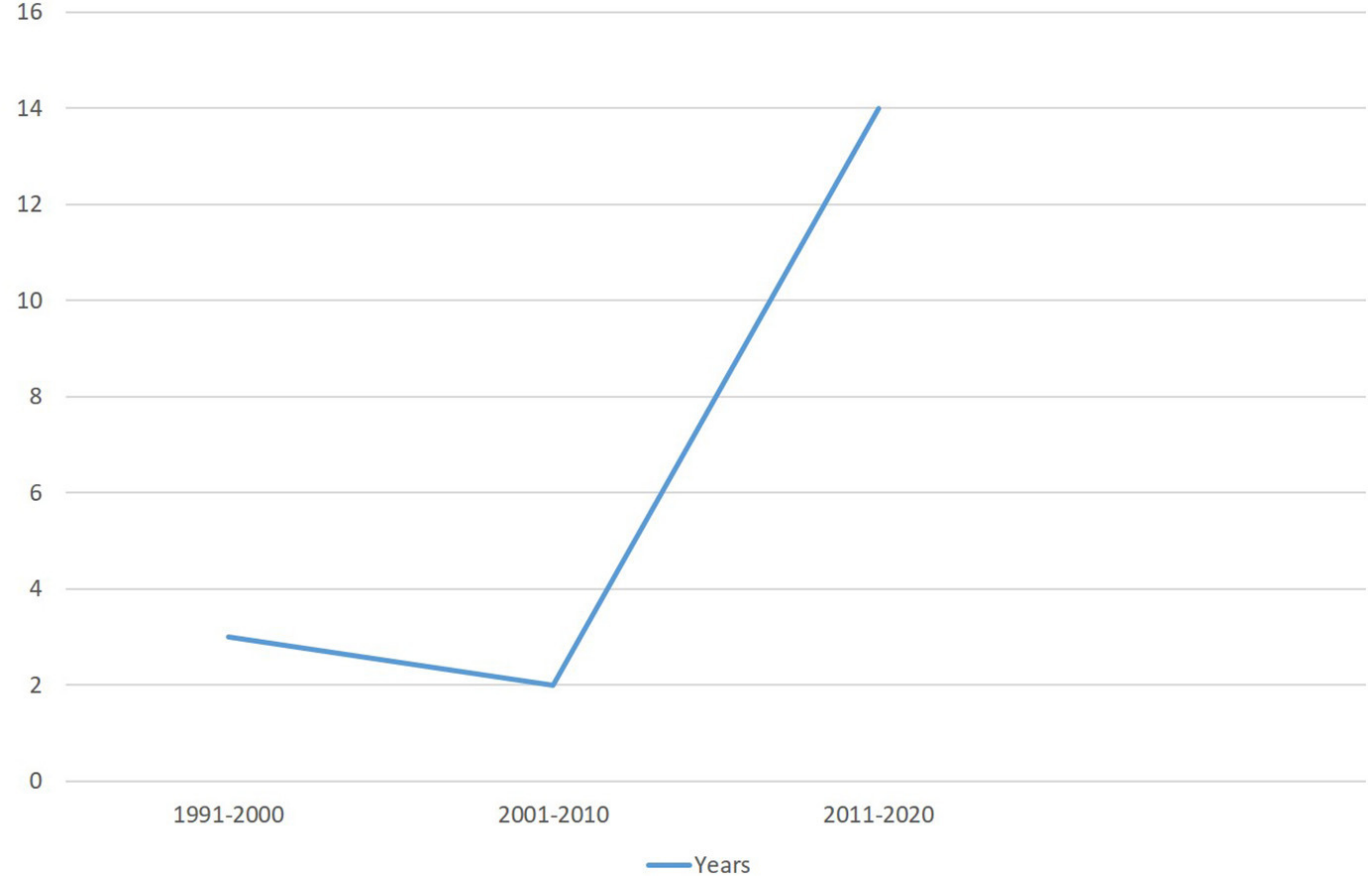
Graph of the number of publications across time.

Most of the included studies were conducted in Europe: two in Italy (Petri et al., 2018; Smorti et al., 2020), two in Germany (Dubber et al., 2015; Matthies et al., 2020), two in the Netherlands (de Cock et al., 2016; Cuijlits et al., 2019), two in Poland (Fijałkowska and Bielawska-Batorowicz, 2019; Zdolska-Wawrzkiewicz et al., 2020), one in France (Luz et al., 2017), and one in Sweden (Hjelmstedt and Collins, 2008). Three studies were carried out in Australia (Condon et al., 2013; Rossen et al., 2016, 2017) and four in the US (Mercer and Ferketich, 1994; Ferketich and Mercer, 1995; Müller, 1996; Damato, 2004). One study was conducted in Turkey (Daglar and Nur, 2018) and one in Iran (Taffazoli et al., 2015). All the papers were published between 1994 and 2020 (Figure 2). Furthermore, only five studies focusing on the relationship between pre-natal attachment and parent-to-infant attachment were published before 2011 (Mercer and Ferketich, 1994; Ferketich and Mercer, 1995; Müller, 1996; Damato, 2004; Hjelmstedt and Collins, 2008). This lapse in research is noteworthy, since the psychological process in which women become emotionally involved and attached to their fetus in pregnancy had already been theorized and linked to the parent-child relationship after birth during the 1950s by several psychoanalytic theorists (Deutsch, 1945; Winnicott, 1956; Benedek, 1958, 1959; Bibring, 1959; Bibring et al., 1961).

### Methodological Issues

All of the studies included in the current systematic review adopted a quantitative approach based on the administration of self-report instruments (Mercer and Ferketich, 1994; Ferketich and Mercer, 1995; Müller, 1996; Damato, 2004; Hjelmstedt and Collins, 2008; Condon et al., 2013; Dubber et al., 2015; de Cock et al., 2016; Rossen et al., 2016, 2017; Luz et al., 2017; Daglar and Nur, 2018; Petri et al., 2018; Cuijlits et al., 2019; Fijałkowska and Bielawska-Batorowicz, 2019; Matthies et al., 2020; Smorti et al., 2020; Zdolska-Wawrzkiewicz et al., 2020). Only one paper resorted to an observational approach during post-partum (Taffazoli et al., 2015). Each behavior was observed and recorded focusing on three groups of attachment behaviors: emotional behaviors, proximity behaviors and caring behaviors (Taffazoli et al., 2015). Finally, one study assessed both pre-natal attachment and parent-to-infant attachment using a quantitative as well as a projective approach (Fijałkowska and Bielawska-Batorowicz, 2019).

Only Luz et al. (2017) analyzed the influence of pre-natal attachment on one's own parent-to-infant attachment as well as on their partner's through dyadic data analysis.

Considering the included participants, the majority of the papers focused on pregnant women (16 studies; Mercer and Ferketich, 1994; Müller, 1996; Damato, 2004; Dubber et al., 2015; Taffazoli et al., 2015; de Cock et al., 2016; Rossen et al., 2016, 2017; Luz et al., 2017; Daglar and Nur, 2018; Petri et al., 2018; Cuijlits et al., 2019; Fijałkowska and Bielawska-Batorowicz, 2019; Matthies et al., 2020; Smorti et al., 2020; Zdolska-Wawrzkiewicz et al., 2020), while only six studies assessed the two variables among fathers (Ferketich and Mercer, 1995; Hjelmstedt and Collins, 2008; Condon et al., 2013; de Cock et al., 2016; Luz et al., 2017; Fijałkowska and Bielawska-Batorowicz, 2019).

All of the studies were conducted on parents who were at least 18 years old. Considering socioeconomic characteristics, most participants had a university or a college degree, were employed, had a middle socioeconomic status, were married or involved in a stable relationship, and belonged to an ethnic majority. Only two studies, conducted in Iran and in Turkey, considered unemployed women or housewives and women with a diploma degree (Taffazoli et al., 2015; Daglar and Nur, 2018). The majority of the studies included both primiparous and multiparous parents (Mercer and Ferketich, 1994; Ferketich and Mercer, 1995; Damato, 2004; Dubber et al., 2015; Rossen et al., 2016, 2017; Luz et al., 2017; Daglar and Nur, 2018; Cuijlits et al., 2019; Matthies et al., 2020; Smorti et al., 2020; Zdolska-Wawrzkiewicz et al., 2020).

Lastly, one study analyzed the relationship between the two variables in fathers with an *in vitro* fertilization (IVF) pregnancy (Hjelmstedt and Collins, 2008) and one paper included a group of women with a twin pregnancy (Damato, 2004).

Differences emerged considering the assessment tools adopted in the included studies. To evaluate maternal pre-natal attachment, the most commonly used tool (seven studies; de Cock et al., 2016; Rossen et al., 2016, 2017; Luz et al., 2017; Daglar and Nur, 2018; Petri et al., 2018; Fijałkowska and Bielawska-Batorowicz, 2019) was the Maternal Antenatal Attachment Scale (MAAS; Condon, 1993). Five papers (Mercer and Ferketich, 1994; Dubber et al., 2015; Taffazoli et al., 2015; Matthies et al., 2020; Zdolska-Wawrzkiewicz et al., 2020) administered the Maternal Fetal Attachment Scale (MFAS; Cranley, 1981). Three studies (Müller, 1996; Damato, 2004; Smorti et al., 2020) used the Pre-natal Attachment Inventory (PAI; Müller, 1993). One study (Cuijlits et al., 2019) administered the Pre- and Post-natal Bondings Scale (PPBS; Cuijlits et al., 2016), and one paper (Fijałkowska and Bielawska-Batorowicz, 2019) applied the Pictorial Representation of Attachment Measure (PRAM; van Bakel et al., 2013).

Considering the assessment of paternal pre-natal attachment, the most commonly used tool (four studies) (Condon et al., 2013; de Cock et al., 2016; Luz et al., 2017; Fijałkowska and Bielawska-Batorowicz, 2019) was the Paternal Antenatal Attachment Scale (PAAS; Condon, 1993). Two studies (Ferketich and Mercer, 1995; Hjelmstedt and Collins, 2008) used the Paternal Fetal Attachment Scale (PFAS; Cranley, 1981) and one paper (Fijałkowska and Bielawska-Batorowicz, 2019) administered the PRAM.

Regarding the assessment of mother-to-infant attachment, most of the studies (seven studies; de Cock et al., 2016; Rossen et al., 2016, 2017; Luz et al., 2017; Petri et al., 2018; Fijałkowska and Bielawska-Batorowicz, 2019; Smorti et al., 2020) administered the Maternal Post-natal Attachment Scale (MPAS; Condon and Corkindale, 1998). Three articles (Dubber et al., 2015; Matthies et al., 2020; Zdolska-Wawrzkiewicz et al., 2020) used the Post-partum Bonding Questionnaire (PBQ-25; Brockington et al., 2001 and PBQ-16; Reck et al., 2006); two (Müller, 1996; Damato, 2004) administered the Maternal Attachment Inventory (MAI; Müller, 1994). Two studies (Mercer and Ferketich, 1994; Müller, 1996) applied the How I Feel About My Baby Now Questionnaire (FAB; Leifer, 1977). One study (Cuijlits et al., 2019) administered the Pre- and Post-natal Bondings Scale (PPBS; Cuijlits et al., 2016); one study (Taffazoli et al., 2015) used the Avant's Questionnaire of Mother-Infant Attachment (Avant, 1982) and one paper (Daglar and Nur, 2018) administered the Mother-to-Infant Bonding Scale (MIBS; Taylor et al., 2005). Finally, one study (Fijałkowska and Bielawska-Batorowicz, 2019) resorted to the PRAM.

To assess father-to-infant attachment, most studies (four studies; Condon et al., 2013; de Cock et al., 2016; Luz et al., 2017; Fijałkowska and Bielawska-Batorowicz, 2019; Zdolska-Wawrzkiewicz et al., 2020) applied the Paternal Post-natal Attachment Scale (PPAS; Condon et al., 2008). One study (Hjelmstedt and Collins, 2008) used the Father Infant Attachment Inventory (FIAI; Hjelmstedt and Collins, 2008), while one paper (Fijałkowska and Bielawska-Batorowicz, 2019) administered the PRAM. Finally, one study (Ferketich and Mercer, 1995) used the FAB.

For a detailed description of the assessment tools used to evaluate parent-to-infant attachment and the dimensions explored see Table 1.

The pre-natal assessment time of the considered variables was homogeneous. In all studies but two, pre-natal attachment tools were administered from the second half of pregnancy, while Rossen et al. (2016, 2017) assessed pre-natal attachment in all the three trimesters of pregnancy. One study did not provide clear information on the assessment time (Zdolska-Wawrzkiewicz et al., 2020). Conversely, the administration time of post-partum tools was heterogeneous. Parent-to-infant attachment was assessed from the post-partum hospitalization period (Mercer and Ferketich, 1994; Ferketich and Mercer, 1995) up to 24 months after childbirth (de Cock et al., 2016).

### Main Findings

All of the studies included in the current systematic review demonstrated that greater levels of pre-natal attachment are associated with better parent-to-infant attachment during post-partum. Specifically, 16 articles found a significant and positive association between pre-natal attachment and parent-to-infant attachment (Mercer and Ferketich, 1994; Ferketich and Mercer, 1995; Müller, 1996; Damato, 2004; Hjelmstedt and Collins, 2008; Condon et al., 2013; Taffazoli et al., 2015; de Cock et al., 2016; Rossen et al., 2016, 2017; Luz et al., 2017; Daglar and Nur, 2018; Petri et al., 2018; Cuijlits et al., 2019; Fijałkowska and Bielawska-Batorowicz, 2019; Smorti et al., 2020), and three studies identified a negative association between pre-natal attachment and post-partum bonding impairments (Dubber et al., 2015; Matthies et al., 2020; Zdolska-Wawrzkiewicz et al., 2020). However, while 18 of the studies included identified a significant relationship between the total scores of the two variables (Mercer and Ferketich, 1994; Ferketich and Mercer, 1995; Müller, 1996; Damato, 2004; Hjelmstedt and Collins, 2008; Condon et al., 2013; Dubber et al., 2015; de Cock et al., 2016; Rossen et al., 2016, 2017; Luz et al., 2017; Daglar and Nur, 2018; Petri et al., 2018; Cuijlits et al., 2019; Fijałkowska and Bielawska-Batorowicz, 2019; Matthies et al., 2020; Smorti et al., 2020; Zdolska-Wawrzkiewicz et al., 2020), Taffazoli et al. (2015) found an association between pre-natal attachment and maternal emotional and proximity behaviors but not with caring ones.

Regardless of the adopted assessment tools and approaches (i.e., self-reports or behavioral observations), the association between pre-natal attachment and parent-to-infant attachment was confirmed in primiparous and multiparous mothers and fathers, both in advantageous (Ferketich and Mercer, 1995; Müller, 1996; Damato, 2004; Hjelmstedt and Collins, 2008; Condon et al., 2013; Dubber et al., 2015; de Cock et al., 2016; Rossen et al., 2016, 2017; Luz et al., 2017; Petri et al., 2018; Cuijlits et al., 2019; Fijałkowska and Bielawska-Batorowicz, 2019; Matthies et al., 2020; Smorti et al., 2020; Zdolska-Wawrzkiewicz et al., 2020) and disadvantageous socio-economic and socio-demographic conditions (Taffazoli et al., 2015; Daglar and Nur, 2018) as well as in expectant parents in normative and at-risk pregnancies - i.e., IVF and twin pregnancies (Damato, 2004; Hjelmstedt and Collins, 2008). However, while 17 studies identified a significant association between the two variables regardless of the post-natal assessment period (i.e., from the 8th day to the 24th month after childbirth), in the study of Mercer and Ferketich (1994) a significant association was found only considering post-partum hospitalization period (and not at 1, 4, and 8 months post-partum) among inexperienced mothers (those with no previous children) and until the 4th month post-partum (i.e., during post-partum hospitalization, at 1 and 4 months, and not at 8 months) among experienced mothers (those with one or more previous children). Furthermore, Ferketich and Mercer (1995) identified a significant association between pre-natal attachment and father-to-infant attachment only until the 4th month post-partum (i.e., during post-partum hospitalization, at 1 and 4 months, and not at 8 months) in experienced fathers and until the 1st month after childbirth (during post-partum hospitalization and at 1 months, and not at 4 and 8 months post-partum) in inexperienced fathers.

In addition to finding a direct association between the two variables among mothers, Smorti et al. (2020) identified a partial mediation through traumatic childbirth experiences. Maternal pre-natal attachment reduced the level of PTSD symptoms associated with traumatic childbirth experiences which in turn promoted a higher quality of mother-to-infant attachment (Smorti et al., 2020). Furthermore, Damato (2004) showed that the association between maternal antenatal attachment and mother-to-infant attachment in twin pregnancies was moderated by post-partum depression, method of delivery and the need for admission to the Neonatal Intensive Care Unit (NICU). Finally, Luz et al. (2017) found that both paternal and maternal pre-natal attachment were significantly associated with father-to-infant attachment. On the contrary, mother-to-infant attachment was not predicted by paternal pre-natal attachment (Luz et al., 2017).

## Discussion

The current systematic review aimed to review and systematize the literature on the association between pre-natal attachment and parent-to-infant attachment, clarifying and discussing the emerging results while reporting relevant information for clinical purposes.

Considering inclusion and exclusion criteria, 19 papers were included. Sixteen studies highlighted a significant and positive association between pre-natal attachment and parent-to-infant attachment, and three articles identified a negative association between antenatal attachment and post-partum bonding disorders. The results were confirmed evaluating parent-to-infant attachment from post-partum hospitalization to 24 months post-partum, including both women and men, in normative and at-risk pregnancies, irrespective of the used assessment approach (i.e., self-report measures, observations, projective measures). Only Taffazoli et al. (2015) identified a positive relation between pre-natal attachment and emotional and proximity behaviors, while they did not confirm these results with respect to caring behaviors. This inconsistent finding may be a consequence of the cultural impact on parental representation of their caregiving role and of their child's characteristics. Indeed, this is the only study conducted in the Middle East among the reviewed papers otherwise European. Overall, these findings highlight that regardless of the assessment approach used to evaluate parent-to-infant attachment, antenatal attachment is a precursor of attachment bond in the early post-partum, considering both affects, cognitions, behaviors and non-verbal representations as indicators. However, only few studies resorted to observational or projective measures, therefore these results have to be cautiously considered and other studies are necessary.

Nonetheless, these findings support the hypothesis that the attachment bond starts to develop during pregnancy through the development of affects, fantasies and mental representations of the unborn child which influence the relationship with the real child after birth. Pre-natal attachment has an impact on the parents' affects and cognitions as well as on their daily interactions with the child during the early post-partum period, promoting the establishment of early, secure and healthy relationships that were found to be associated with more positive outcomes in the child's development and post-natal infant-to-parent attachment (Sroufe, 2005; Murphy and Laible, 2013; Zimmer-Gembeck et al., 2017; Matthies et al., 2020).

However, several studies identified only low or moderate associations between pre-natal attachment and parent-to-infant attachment (e.g., Müller, 1996; Damato, 2004; de Cock et al., 2016; Rossen et al., 2016, 2017; Cuijlits et al., 2019; Matthies et al., 2020). These data may in part depend on the applied assessment tool; not only, other factors may also influence pre-natal and early post-partum attachment, and their link. In line with these findings, some studies (Damato, 2004; Smorti et al., 2020) included in the current systematic review observed that the relationship between antenatal attachment and parent-to-infant attachment was mediated or moderated by gestational childbirth experiences (i.e., cesarean delivery; traumatic childbirth experiences), as well as by the need for neonatal intensive care and psychological symptoms (i.e., post-partum depression). Thus, other perinatal variables seem to intervene in the association between pre-natal and parent-to-infant attachment, and more studies are necessary to clarify these preliminary findings, including individual, relational and contextual variables.

Although the association was widely confirmed among mothers, few studies have been conducted on fathers (Ferketich and Mercer, 1995; Hjelmstedt and Collins, 2008; Condon et al., 2013; de Cock et al., 2016; Luz et al., 2017; Fijałkowska and Bielawska-Batorowicz, 2019). For men, the development and establishment of an attachment relationship with the unborn child presents greater complexities due to the absence of physical evidences and bodily experiences (Habib and Lancaster, 2005, 2010; Righetti et al., 2005; Ustunsoz et al., 2010; Della Vedova and Burro, 2017). Accordingly, lower levels of pre-natal attachment were found among men compared with women in several studies (Steen et al., 2012; Vreeswijk et al., 2014; Della Vedova and Cristini, 2019; Fijałkowska and Bielawska-Batorowicz, 2019) with paternal pre-natal attachment being influenced by the father's perception of the partner's attitude toward him during pregnancy and his perceived distress (Della Vedova and Cristini, 2019). Furthermore, as highlighted in the current systematic review, fathers with a higher pre-natal attachment and whose partners have higher levels of maternal pre-natal attachment show a higher father-to-infant attachment in the first post-natal period (Luz et al., 2017). This suggests that the development and the stability of the attachment relationship in fathers is influenced by individual and relational variables (Sandberg and Hofferth, 2001; Yeung et al., 2001; Fijałkowska and Bielawska-Batorowicz, 2019), such as the quality and intensity of his partner's attachment to the fetus, highlighting the role of women during the perinatal period for the assumption of the paternal identity (Ferketich and Mercer, 1995; Buist et al., 2003; Condon et al., 2004, 2013; Habib and Lancaster, 2010; Luz et al., 2017). However, due to the scarcity of studies on the subject, it is necessary to increase studies on male samples to strengthen and deepen these conclusions.

Furthermore, most of the studies were conducted on specific groups of participants such as parents who were married or involved in a stable relationship, with a college degree, employed, with a middle-class socio-economic status and belonging to an ethnic majority, and parents who experienced normative pregnancies. Therefore, the generalizability of these results is limited.

### Clinical Implications

Since all of the studies confirmed the association between pre-natal attachment and parent-to-infant attachment, and taking into account that parent-to-infant attachment can impact the well-being of the family system (e.g., the child's development and emotional regulation, parental responsiveness and sensitivity to the child's needs, mother-child interactions), regular screening processes, and preventive programs are suggested at an early stage of pregnancy aiming to increase the strength of the pre-natal attachment and to indirectly influence the parent-child relationship after birth (Brandon et al., 2009; Young, 2013; Parfitt et al., 2014; Cataudella et al., 2016). Parental–fetal attachment can be modifiable by specific supporting interventions that emerged as effective (Brisch et al., 2003; Brecht et al., 2012; Akbarzade et al., 2014; Cunen et al., 2017; Ekrami et al., 2019; Parlakian and Kinsner, 2019). These programs - such as psychosomatically oriented antenatal classes, home visiting interventions or pre-natal psychoeducation programs - support the development of the attachment bond by providing a psychic space for parenting, promote the parents' awareness of the presence and needs of the child, encourage parents' fantasies about their unborn baby and favor the parent's availability toward their child, perceived as a separate psychological being (Slade, 2005; Ammaniti et al., 2006; Vismara et al., 2020), providing support along the whole perinatal period (Cranley, 1981; Feldman, 2007, 2012).

### Strengths and Limitations

This review presents several limitations. Firstly, statistical conclusions on the results of this systematic review cannot be drawn as it is not a meta-analysis.

Moreover, this review only included papers published in English, thus excluding results published in other languages that could give a broader understanding of the association between pre-natal attachment and parent-to-infant attachment.

According with the inclusion and exclusion criteria, we only focused on the association between pre-natal attachment and parent-to-infant attachment. However, in future systematic reviews it could be useful to consider the association between pre-natal attachment and infant-to-parent attachment, in order to explore long-term effects of antenatal attachment.

Lastly, we did not consider other factors such as parental mentalization and parental representations which could provide a deeper understanding of the variables that influence relationship quality during the post-partum period.

### Future Research Directions

Firstly, further research would be useful to deeply analyze the association between pre-natal attachment and parent-to-infant attachment in male samples and in non-normative pregnancies.

Furthermore, since some studies (Damato, 2004; Smorti et al., 2020) highlighted the role of mediators (i.e., level of PTSD symptoms linked to childbirth) and moderators (i.e., depression, method of delivery and the need for the admission to the NICU) in the association between pre-natal attachment and parent-to-infant attachment, and considering the low or moderate association between the two variables found in many studies (e.g., Müller, 1996; Damato, 2004; de Cock et al., 2016; Rossen et al., 2016, 2017; Cuijlits et al., 2019; Matthies et al., 2020), it could be useful to test more complex models to clarify mechanisms through which pre- and parent-to-infant attachment are connected, and risk and protective factors involved (Sameroff and Fiese, 2000). Future studies investigating the role of parental mentalization in pregnancy as well as the unconscious level of parental representations of caregiving and the child are needed, in order to reach a broader comprehension of psychological and cognitive factors that can influence the relationship quality during early post-partum.

Most of the samples included participants belonging to an ethnic majority, with a high education, medium socioeconomic status, employed and with a stable relationship (Mercer and Ferketich, 1994; Ferketich and Mercer, 1995; Müller, 1996; Damato, 2004; Dubber et al., 2015; Taffazoli et al., 2015; de Cock et al., 2016; Rossen et al., 2016, 2017; Luz et al., 2017; Daglar and Nur, 2018; Petri et al., 2018; Cuijlits et al., 2019; Fijałkowska and Bielawska-Batorowicz, 2019; Matthies et al., 2020; Smorti et al., 2020; Zdolska-Wawrzkiewicz et al., 2020). On the contrary, very few studies considered samples with socio-economic and demographic disadvantages (Taffazoli et al., 2015; Daglar and Nur, 2018). Further research that include different socio-demographic contexts are necessary for a better generalizability of the above results. Additionally, adopting a cross-cultural design in future research would allow to investigate the presence of homogeneity and heterogeneity between different countries and cultures (Taffazoli et al., 2015; Daglar and Nur, 2018), considering that attitudes toward the unborn child and child-rearing patterns differ on the basis of nationality or cultural status (Omani samani et al., 2016; Salehi et al., 2018; Zaidman-Mograbi et al., 2020).

Finally, it is noteworthy that only one study (Taffazoli et al., 2015) used an observational approach, while the remaining papers applied self-report measures, focusing mainly on affective and cognitive indicators of parent-to-infant attachment. Condon and Corkindale (1998) highlighted that behavioral observations of attachment in the early post-partum period can be expensive in research and clinical setting, biased by situational factors and social desirability, and unable to capture the subjective experience of parent-to-infant attachment, recommending using self-report questionnaires able to identify the experiential dimension of attachment. However, combining these two assessment procedures may strengthen the reached conclusions, and may deepen how the affective experience of attachment both during pregnancy and in the post-partum is translated in caregiving behaviors in the early post-natal period. Thus, in future studies it would be important to assess parent-to-infant attachment using both self-report measures and observational procedures (Condon and Corkindale, 1998; Condon, 2012).

## Conclusion

This systematic review on the association between pre-natal attachment and parent-to-infant attachment found a significant association between the two variables. However, few results emerged on male samples, non-normative pregnancies and on disadvantageous socio-economic and demographic samples, suggesting that for these participants results should be interpreted with caution. Future studies are needed to better clarify this relationship and generalize these results.

Nonetheless, these findings can be useful for clinical purposes, providing information for the implementation of screening processes and interventions aimed at enhancing the attachment relationship starting from pregnancy, improving the parent-child relationship and familial well-being during post-partum.

## Author Contributions

TT and LR took overall responsibility for the creation of the framework used in this review and the selection of the papers. MG, FS, LV, and AMDV searched for the articles discussed in the review. TT and LR supervised the entire work. All authors were involved in the discussion, the writing, and the revision of the manuscript, and they gave the final approval of the version to be published.

## Conflict of Interest

The authors declare that the research was conducted in the absence of any commercial or financial relationships that could be construed as a potential conflict of interest.
